# Synthesis of MoIn_2_S_4_@CNTs Composite Counter Electrode for Dye-Sensitized Solar Cells

**DOI:** 10.1186/s11671-020-03410-0

**Published:** 2020-09-21

**Authors:** Gentian Yue, Renzhi Cheng, Xueman Gao, Leqing Fan, Yangfan Mao, Yueyue Gao, Furui Tan

**Affiliations:** 1 Henan Key Laboratory of Photovoltaic Materials and Laboratory of Low-Dimensional Materials Science, Henan University, Kaifeng, 475004 People’s Republic of China; 2 Fujian Key Laboratory of Functional Materials, Huaqiao University, Xiamen, 361021 Fujian People’s Republic of China; 3 School of Chemical Engineering and Materials Science, Quanzhou Normal University, Quanzhou, 362000 People’s Republic of China

**Keywords:** Counter electrode, Dye-sensitized solar cell, MoIn_2_S_4_, Carbon nanotubes, Power conversion efficiency

## Abstract

A ternary and composite MoIn_2_S_4_@CNTs counter electrode (CE) with a hedgehog ball structure was synthesized by using a facile one-step hydrothermal method. The composite MoIn_2_S_4_@CNTs film possesses large specific surface area through N_2_ adsorption-desorption isotherms test, which is advantageous to adsorb more electrolyte and provide larger active contact area for the electrode. In addition, the composite MoIn_2_S_4_@CNTs CE exhibits low charge transfer resistance and fine electrocatalytic ability made from a series of electrochemical tests including cyclic voltammetry, electrochemical impedance, and Tafel curves. Under optimal conditions, the DSSC based on the MoIn_2_S_4_@CNTs-2 composite CE achieves an impressive power conversion efficiency as high as 8.38%, which remarkably exceeds that of the DSSCs with the MoIn_2_S_4_ CE (7.44%) and the Pt electrode (8.01%). The current work provides a simplified preparation process for the DSSCs.

## Background

In recent decades, it is urgent to exploit and utilize renewable energy substituting the conventional fossil fuels with the severe energy shortage and environmental degradation increasing [1, 2]. Dye-sensitized solar cell (DSSC) has attracted wide-spread research by virtue of its environmental friendliness, facile preparation process, brilliant photovoltaic performance, and so on [3, 4]. The counter electrode (CE), as one of the significant components of a DSSC, plays the role of gathering electrons from external circuit and catalyzing the reduction reaction of I_3_^−^ to I^−^ in the liquid electrolyte [5, 6]. Generally, ideal CE materials contain the merits of high electrical conductivity and remarkable catalytic activity. However, platinum (Pt) as a prevalent and efficient CE material is confined to large-scale commercialized application because of the major weaknesses of scarcity, expensiveness, and poorly long-term stability [7, 8]. Therefore, a great many of efforts have been made to develop easily accessible, cost-effective, and Pt-like catalytic activity applied in DSSC for years [9].

Up to now, various kinds of outstanding alternative materials have been proposed, such as carbonaceous materials [10, 11], transition metal chalcogenides [12], conductive polymers [13], metal alloys [14], and their compounds [15, 16]. Among them, the binary transition metal chalcogenides attracted a great deal of attention due to their unique structure and chemical properties. For instance, the synthesized MoS_2_ on FTO substrate exhibits a sandwich-layered structure, larger surface area, and more active edge sites leading to extremely photoelectric performance as a CE for DSSC [17]. Meanwhile, extensive research work focused on the catalytic activity for I_3_^−^ reduction also has been made for WS_2_ [18], FeS_2_ [19], CoS [20], and NiS_2_ [21], which were comparable to or even better than that of the Pt electrode. Nevertheless, the inherent characteristic of these materials, such as low electrical conductivity and only two-fixed chemical compositions, hampered further improvement in their catalytic activity [22]. Hence, numerous methods aimed to overcome the abovementioned shortcoming were adopted to synthesize multinary transition metal chalcogenides by component elements adjusting, structure designing, and morphology tailoring. Fortunately, considerable multinary transition metal chalcogenides have achieved significant enhancements in catalytic ability for DSSCs, such as NiCo_2_S_4_ [23], MIn_2_S_4_ (M = Fe, Co, Ni) [22], CuInS_2_ [24], CoCuWS_*x*_ [25], and Ag_8_GeS_6_ [26], which catalytic ability are obviously much better than that of their binary counterparts.

Moreover, it is widely acknowledged that carbon nanotubes (CNTs) exhibit considerably novel characteristics of large specific surface area, superb electrical conductivity, high mechanical strength, and photochemical stability, which are widely used in the synthesis and modification of other materials [27]. Regrettably, CNTs show poor electrocatalytic activity for I_3_^−^ reduction, which greatly limits their application independently in a DSSC device. Fortunately, a large number of studies have shown that the composite CE modified with CNTs all obtained greatly improved photoelectric performance for DSSCs [18, 28, 29]. Liu et al. have reported a flower-like hierarchical structure of Cu_2_MnSnS_4_/CNT (CMTS/CNT) CE via solvothermal method in DSSC gained a photoelectric conversion efficiency of 8.97%, much higher than that of the DSSCs with CMTS (6.21%) and Pt (8.37%) CEs [29].

Based on the above considerations, in this study, a MoIn_2_S_4_@CNT composite CE of DSSC with hedgehog ball structure was synthesized by using a facile one-step hydrothermal method and expected to improve higher device performance. Scanning electron microscope results show that different CNT contents result in visible changes on morphology. According to a series of electrochemical characterizations including cyclic voltammetry (CV), electrochemical impedance spectroscopy (EIS), and Tafel curves tests, the MoIn_2_S_4_@CNTs CE indicates a remarkable catalytic activity and fine charge transfer resistance. The DSSC assembled with the MoIn_2_S_4_@CNTs CE with suitable content achieves a superior power conversion efficiency of 8.38%, which is better than that of the DSSC based on the Pt CE (8.01%).

## Methods

### Materials

Sodium molybdate dihydrate (Na_2_MoO_4_·2H_2_O), indium chloride tetrahydrate (InCl_3_·4H_2_O), and thioacetamide (TAA) were purchased from Shanghai Chemical Agent Ltd., China, which were used directly without further purification. Carbon nanotubes (CNTs) were gained from Aladdin Chemical Agent Ltd., China. The commercial Z907 dye was obtained from Solaronix Ltd. (Switzerland). The fluorine-doped SnO_2_ (FTO) glass, purchased from NSG, Japan (15 Ω sq^−1^), were cleaned with detergent and acetone as well as ethyl alcohol in sequence after cutting into squares of 1.5 cm × 2.0 cm.

### Preparation of porous TiO_2_ photoanodes

The colloid of TiO_2_ was prepared as our previous work [30]. The dye-sensitized TiO_2_ photoanodes were fabricated as follows: Firstly, 3-M tape (50 μm thick) with exposed area of 0.283 cm^2^ was attached on FTO. Subsequently, the as-prepared TiO_2_ colloid was coated on by using a blade-coating method. Secondly, the drying TiO_2_ electrode was sintered at 450 °C for 30 min in muffle furnace. Afterwards, the TiO_2_ electrode was immersed in a 40-mM titanium tetrachloride (TiCl_4_) aqueous solution at 70 °C for 30 min, and then annealed in air at 450 °C for 30 min. After cooling down to room temperature, the TiO_2_ electrode was immersed in dye Z907 (0.3 mM) absolute ethanol solution for 24 h to adsorb sufficient dyes and obtained the resultant dye-sensitized TiO_2_ photoanode.

### Fabrication of ternary MoIn_2_S_4_@CNTs CE

The MoIn_2_S_4_ thin films were grown directly onto FTO substrates by a simple approach referring to our previous report [31]. In a typical preparation, 0.0696 g Na_2_MoO_4_·2H_2_O, 0.169 g InCl_3_·4H_2_O, and 0.1394 g TAA were diffused in 30 ml deionized water under ultrasonicating for 2 h until all the reactants were dissolved. The pre-cleaned FTO substrates were put into a 100-ml Teflon-lined stainless steel autoclave with the conductive side facing up before transferring the above precursor mixture into it. After being sealed, the autoclave was placed in an oven and heated under 200 °C for a reaction time of 15 h. The FTO glass substrates covered with MoIn_2_S_4_ materials were taken out from the autoclave, washed with ethanol, deionized water, and then dried in air under 60 °C for 12 h.

In order to study the impact of the CNT contents on the fabricated composite CE and the performance of the DSSC, different contents of CNTs adding to the precursor were conducted, including three samples in which the amount of CNTs were 10, 20, and 30 mg, respectively, keeping the other reagents and fabrication processes unchanged. The above samples were marked MoIn_2_S_4_ (0 mg), MoIn_2_S_4_@CNTs-1 (10 mg), MoIn_2_S_4_@CNTs-2 (20 mg), and MoIn_2_S_4_@CNTs-3 (30 mg).

For comparison, a pyrolysised Pt CE was employed as the reference CE. The H_2_PtCl_6_ in isopropanol solution (0.50 wt%) was dropped onto the surface of the FTO glass, and then sintered at 450 °C in muffle furnace for 30 min to fabricate the Pt CE.

### Fabrication of the DSSCs

The DSSCs with sandwich structure were constructed by clipping the sample CEs (including MoIn_2_S_4_, various MoIn_2_S_4_@CNTs, and Pt CEs) together with as-prepared dye-sensitized TiO_2_ photoanode. Surlyn was used as spacer between the electrodes and followed by filling the interspace with liquid redox electrolyte that consisted of 0.60 M tetrabutylammonium iodide, 0.10 M lithium iodide, 0.05 M iodine, and 0.50 M 4-tert-butyl-pyridine acetonitrile solution.

### Characterizations

Chemical element composition of the samples was characterized by using X-ray photoelectron spectroscopy (XPS) analysis (Kratos Axis Ultra). The morphological features of samples were observed by field emission scanning electron microscopy (FESEM, JSM-7001F). BET-specific surface area method was employed using a JW-K analyser by nitrogen absorption to test the surface area and pore size distribution. The other relevant electrochemical properties were researched by a CHI660E electrochemical workstation. The electrochemical impedance spectroscopy (EIS) was performed in a frequency range of 0.1–10^5^ Hz with a disturbed amplitude of 5 mV. The photovoltaic performances of the DSSCs were carried out by measuring current density-voltage (*J*-*V*) characteristic curves under irradiation of 100 mW cm^−2^ from the solar simulator (CEL-S500, Beijing China Education Au-light Co., Ltd).

## Results and Discussions

### Composition and Morphology

**Fig. 1:**
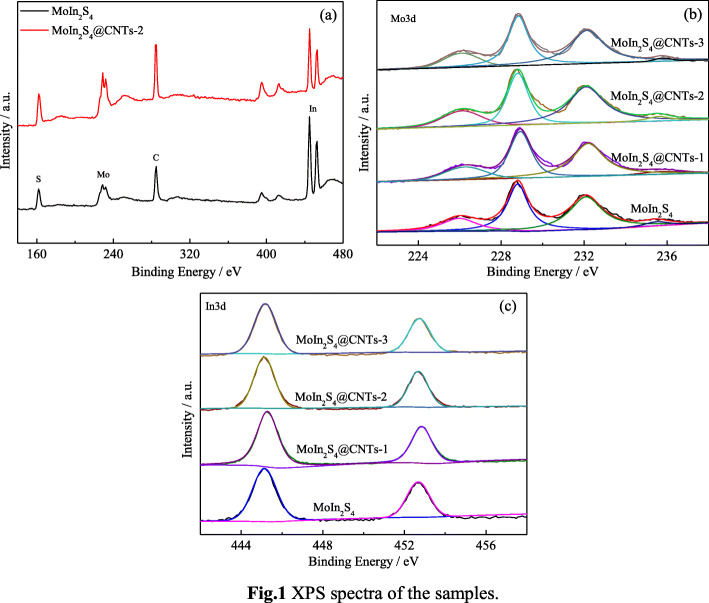
XPS spectra of the samples

The XPS is employed to examine the surface compositions and chemical states of each element in MoIn_2_S_4_ and MoIn_2_S_4_@CNTs films. The spectrum of survey data in MoIn_2_S_4_ and MoIn_2_S_4_@CNTs-2 samples is demonstrated in Fig. 1a to verify the presence of Mo, In, S, and C (as a reference) elements. In addition, all spectra of the four samples, calibrated by the C 1s peak at the binding energy of 284.6 eV [17], are analyzed via the Gaussian fitting method showing in Fig. 1b and c. The C 1s peak appeared in MoIn_2_S_4_ sample is well known to have originated from the adventitious carbon caused by exposure to the air. In the Mo 3d region, two major peaks at 228.8 and 232.1 eV are assigned to Mo 3d5/2 and Mo 3d3/2 of the MoS_2_ [32], respectively. This result confirms that Mo element is in its IV oxidation state, which is reduced to Mo^4+^ (MoIn_2_S_4_) from Mo^6+^ (Na_2_MoO_4_) [33]. The doublet peaks at binding energies of 445.2 and 452.2 eV correspond to In 3d5/2 and In 3d3/2 [24, 34]. As for the XPS spectra of S 2p, the peaks located at 161.8 and 163.1 eV belong to S 2p3/2 and 2p1/2, respectively, which is ascribed to the S_2_^−^ [17, 32]. The above results are well in agreement with our previous studies [31]. Moreover, no other element or extra peak is found in the survey, which reconfirms that the samples synthesized have the similar chemical compositions and structure.

**Fig. 2:**
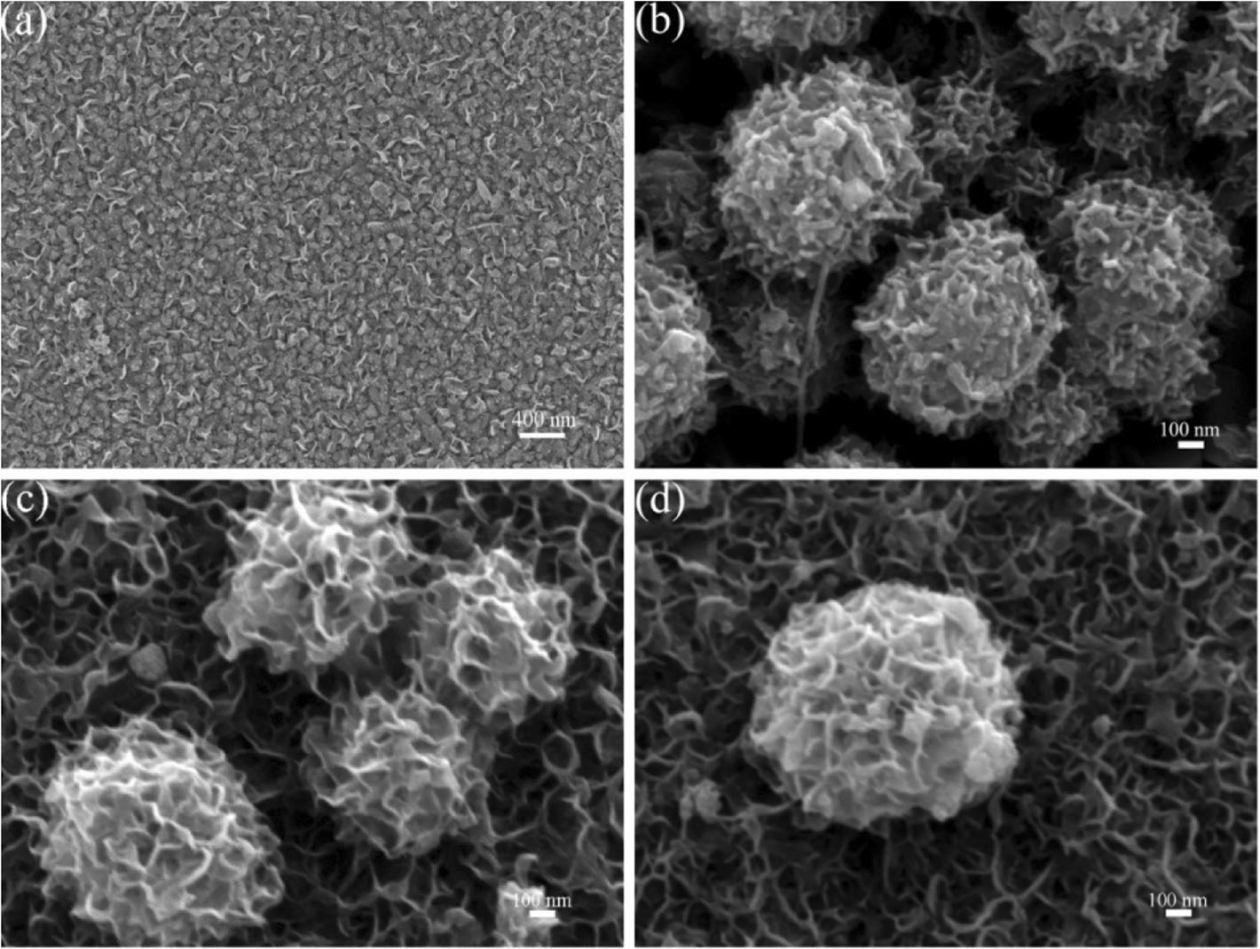
SEM images of **a** MoIn_2_S_4_, **b** MoIn_2_S_4_@CNTs-1, **c** MoIn_2_S_4_@CNTs-2, and **d** MoIn_2_S_4_@CNTs-3

The surface morphologies of as-prepared MoIn_2_S_4_ and MoIn_2_S_4_@CNTs nanofilms are observed by SEM images in Fig. 2. In Fig. 2a, MoIn_2_S_4_ sample displays a petal-like nanosheet structure with a uniform, smooth, and dense surface. Unlike MoIn_2_S_4_ nanofilms, the hedgehog ball structure is found in MoIn_2_S_4_@CNT samples in Fig. 2b–d, and the average diameter of the MoIn_2_S_4_@CNTs nanospheres is around 890 nm. It is easy to observe that so many network nanosheets are fully grown on the FTO substrate. Compared to the MoIn_2_S_4_@CNT samples with low (MoIn_2_S_4_@CNTs-1) and high (MoIn_2_S_4_@CNTs-3) CNT contents, the sample with moderate content (MoIn_2_S_4_@CNTs-2) exhibits more hedgehog balls and nanosheets array on the network of the MoIn_2_S_4_. A good contact performance between the network nanosheet array structure with large number hedgehog balls on FTO substrate facilitates the reduction of I_3_^−^ attributed to its good conductivity and vast catalytic active sites, which can be predicted that the MoIn_2_S_4_@CNTs-2 CE will achieve better performance than that of the MoIn_2_S_4_, MoIn_2_S_4_@CNTs-1, and MoIn_2_S_4_@CNTs-3 CEs. Moreover, dissimilar morphologies between MoIn_2_S_4_ and MoIn_2_S_4_@CNTs indicate that CNTs play a pivotal role in controlling morphology of the samples.

**Fig. 3:**
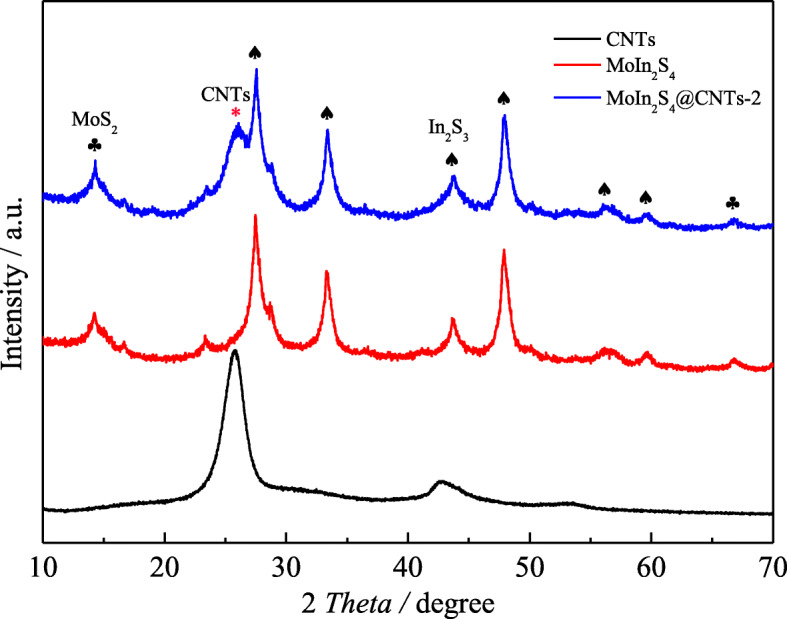
XRD patterns of various samples

Figure 3 shows the XRD patterns of various samples. Among them, the diffraction peaks at 25.74 and 42.85° attribute to the signals of the CNTs [31]. The diffraction peaks at 2θ = 27.5, 33.4, 43.7, 47.9, 56.2, and 59.6° belong to the (311), (400), (511), (440), (533), and (444) crystallographic plane (JCPDS card no. 32-0456) of In_2_S_3_. The peaks at 14.4 and 66.5° are regarded to (002) and (114) crystal planes of cubic structure (JCPDS card no. 37-1492) for the MoS_2_ [33]. From the MoIn_2_S_4_@CNTs-2 and MoIn_2_S_4_ samples, the peaks of the above discussed appear well in the both samples. Compared to the MoIn_2_S_4_ sample, the peak for the CNTs at 25.74° can be seen clearly in MoIn_2_S_4_@CNTs-2 XRD patterns. Thus, it can be inferred that the MoIn_2_S_4_@CNTs-2 materials are synthesized successfully and there are no impurities introduction.

**Fig. 4:**
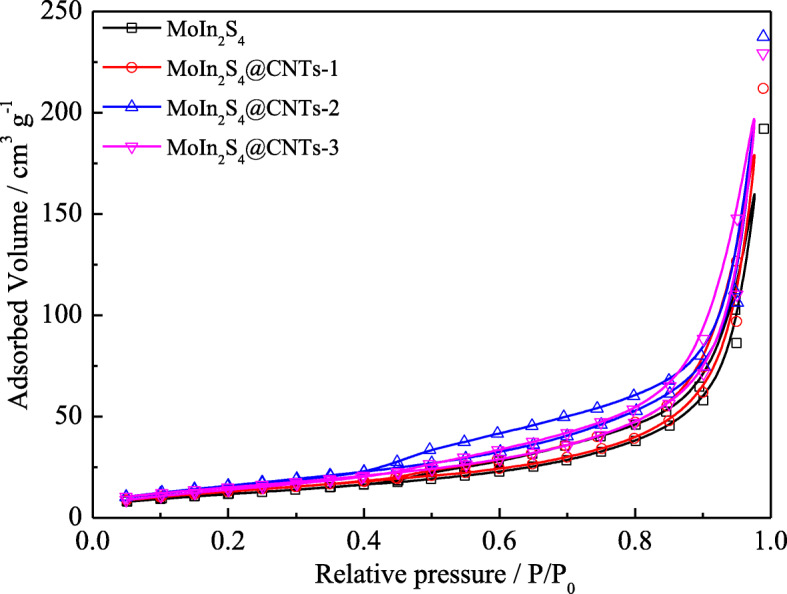
N_2_ absorption-desorption isotherms of various samples

N_2_ adsorption-desorption isotherms are measured and showed in Fig. 4 to explore the specific surface areas and pore features. Generally, the larger specific surface area facilitates more convenient charge transmission on the CE/electrolyte interface [35]. It demonstrates from Fig. 4 that the samples possess an evident hysteresis loop of type IV adsorption-desorption behavior, and their corresponding data calculated from the Brunauer-Emmett-Teller (BET) and Barrett-Joyner-Halenda (BJH) method are tabulated in Table 1. By comparison, it is easy to find that the specific surface area and average pore diameter of the MoIn_2_S_4_@CNT samples are much better than that of the MoIn_2_S_4_. Among the three MoIn_2_S_4_@CNT samples, MoIn_2_S_4_@CNTs-2 shows the largest specific surface area of 66.80 m^2^ g^−1^ and the smallest average pore diameter of 17.8 nm, which can be ascribed to the excellent hedgehog ball structure after doping of moderate CNTs. It is reasonable to believe that the MoIn_2_S_4_@CNTs-2 CE will obtain the fine catalytic activity and hence achieve highly efficient device performance.

**Table 1:** The parameters of N_2_ absorption-desorption isotherms of various samples

CEs	*S*_BET_ (m^2^ g^−1^)	*d*_A_ (nm)	|*J*_pc_| (mA cm^−2^)
MoIn_2_S_4_	44.27	35.9	4.31
MoIn_2_S_4_@CNTs-1	55.79	23.8	4.68
MoIn_2_S_4_@CNTs-2	66.80	17.8	7.47
MoIn_2_S_4_@CNTs-3	61.80	21.2	5.09

### Electrochemical properties

**Fig. 5:**
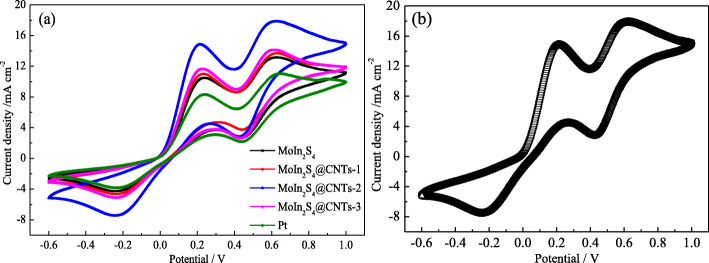
**a** CVs of the various CEs at a scan rate of 60 mV s^−1^. **b** 50 cycles CV of the MoIn_2_S_4_@CNTs-2 CE

CV measurements are carried out to study the electrocatalytic behavior of the as-obtained samples in the potential range of − 0.6 to 1.0 V at a scan rate of 60 mV s^−1^ for the MoIn_2_S_4_ and MoIn_2_S_4_@CNTs CEs, and the calculated values are summed up in Table 1. The left peaks of the two pairs of oxidation and reduction peaks in each CV curve in Fig. 5a are ascribed to the equation (I_3_^−^ + 2e^−^ ↔ 3I^−^), which determines the performance of electrocatalytic activity of the CE materials, especially in DSSCs [23, 36]. The values of the negative reduction peak current density (*J*_pc_), a key parameter in CV test, follows the orders of Pt (3.80 mA cm^−2^) < MoIn_2_S_4_ (4.31 mA cm^−2^) < MoIn_2_S_4_@CNTs-1 (4.68 mA cm^−2^) < MoIn_2_S_4_@CNTs-3 (5.09 mA cm^−2^) < MoIn_2_S_4_@CNTs-2 (7.47 mA cm^−2^). Evidently, the MoIn_2_S_4_ CE itself has good catalytic activity, and the MoIn_2_S_4_@CNTs-2 CE exhibits much higher *J*_pc_ than that of the Pt, MoIn_2_S_4_ CEs, and the other two kinds of MoIn_2_S_4_@CNTs CEs attributed to its distinctive surface morphology, the synergistic effect of CNTs doped, and the larger surface area. The results indicate that the MoIn_2_S_4_@CNTs-2 CE has fine electrocatalytic activity for the I^−^/I_3_^−^ redox couple in DSSC CEs. Figure 5b shows the CV curves of the MoIn_2_S_4_@CNTs-2 CE at the scan rate of 60 mV s^−1^ and they are almost no changing with 50 cycles, which indicates that the MoIn_2_S_4_@CNTs-2 CE possesses excellent electrochemical stability.

**Fig. 6:**
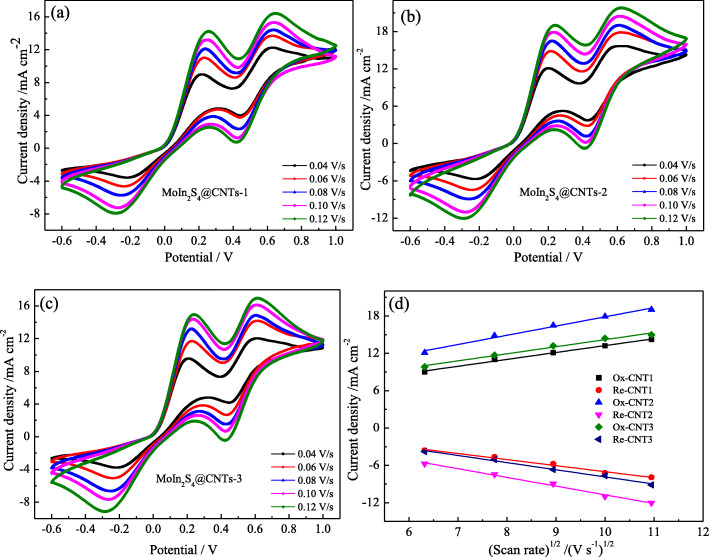
CVs of the MoIn_2_S_4_@CNTs CEs at different scan rates **a** MoIn_2_S_4_@CNTs-1, **b** MoIn_2_S_4@_CNTs-2, **c** MoIn_2_S_4_@CNTs-3, and **d** the influences of the scan rate on the redox peaks current density

Figure 6a–c show the CVs of MoIn_2_S_4_@CNTs CEs at different scan rates. With the scan rates increasing from 20 to 120 mV s^−1^, the oxidation and reduction peaks shift towards positive and negative direction owing to the fast diffusions of I^−^/I_3_^−^ redox couple on the surfaces of CEs and the large electrochemical polarization [28]. Furthermore, Fig. 6d shows the relationship between the anodic and cathodic peaks current densities of the left peak pairs against the square root of sweep rates. The well-fitted linear relations indicate that the redox reaction of I^−^/I_3_^−^ is dominated by diffusion-controlled ion transport [22, 25].

**Fig. 7:**
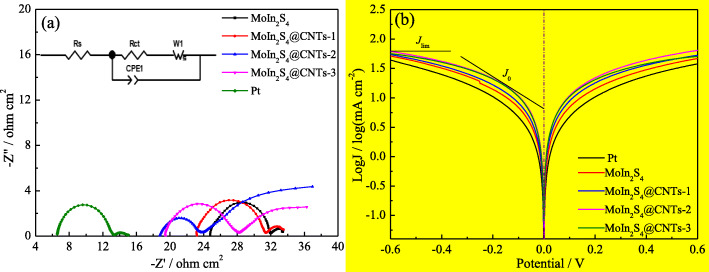
**a** EIS and **b** Tafel curves of the symmetrical MoIn_2_S_4_, MoIn_2_S_4_@CNTs-1, MoIn_2_S_4_@CNTs-2, and MoIn_2_S_4_@CNTs-3 CEs

Figure 7a shows the Nyquist plots of the different samples to further insight into the kinetics of the interfacial charge transfer process. The EIS data made from the fitted curves with a model equivalent circuit of the insert is listed in Table 2. Typically, the Nyquist plots contain two semicircles, the first semicircle on the left represents the charge transfer resistance *R*_ct_ at the CE and electrolyte interface, and the second semicircle corresponds to the Nernst diffusion impedance in the electrolyte, while the intercept of the curve in the high-frequency region on the real axis is known as the series resistance *R*_s_. In general, *R*_s_ and *R*_ct_ are two vital parameters for evaluating the catalytic activity of CE in DSSC. A small *R*_s_ indicates good contact between the catalyst and the substrate, and hence the resistance of the entire device is also small [29, 37, 38]. Meanwhile, little *R*_ct_ stands for a high charge transfer rate. As presented in Table 2, the *R*_s_ values of the MoIn_2_S_4_, MoIn_2_S_4_@CNTs-1, MoIn_2_S_4_@CNTs-2, and MoIn_2_S_4_@CNTs-3 CEs are 24.77, 23.16, 18.96, and 19.58 Ω cm^2^, respectively. Obviously, all the composite MoIn_2_S_4_@CNTs CEs have the smaller *R*_s_ than that of the MoIn_2_S_4_ CE, indicating that the conductivity of the MoIn_2_S_4_@CNTs CEs is enhanced after doping CNTs. Furthermore, among the four CEs, the trends of *R*_ct_ is MoIn_2_S_4_ > MoIn_2_S_4_@CNTs-1 > MoIn_2_S_4_@CNTs-3 > MoIn_2_S_4_@CNTs-2, which suggests an inverse order of electrochemical impedance and catalytic ability of the CEs. MoIn_2_S_4_@CNTs-2 CE possesses the lowest *R*_ct_ value can be attributed to the synergy of the CNTs with fine conductivity and MoIn_2_S_4_ with excellent catalytic ability, resulting in more effective reduction of triiodide on the CE/electrolyte interface. Apparently, the conductivity and catalytic ability of the MoIn_2_S_4_@CNTs composite CEs has been improved greatly than that of the MoIn_2_S_4_ CE, and the result is completely consistent with the BET and CV tests.

**Table 2:** Electrochemical parameters for the different CEs

Electrodes	*R*_s_ (Ω cm^2^)	*R*_ct_ (Ω cm^2^)	*J*_lim_ (mA cm^−2^)	*J*_0_ (mA cm^−2^)
MoIn_2_S_4_	24.77	7.291	1.67	1.11
MoIn_2_S_4_@CNTs-1	23.16	8.078	1.70	1.18
MoIn_2_S_4_@CNTs-2	18.96	3.162	1.81	1.35
MoIn_2_S_4_@CNTs-3	19.58	6.401	1.72	1.32
Pt	6.56	7.177	1.58	1.04

The Tafel polarization curves of the various CEs are measured as shown in Fig. 7b, and the corresponding parameters values are summarized in Table 2. Normally, a standard Tafel curve includes two significant parameters named exchange current density (*J*_0_) and limiting diffusion current density (*J*_lim_). *J*_0_ is related to the catalytic reduction reaction. The larger the *J*_0_ is, the better the catalytic effect. *J*_lim_ is also positively related to the diffusion efficiency of electrolyte. The larger *J*_lim_ indicates the faster diffusion of I_3_^−^ ions [29, 37]. As presented in Fig. 7b and Table 2, the *J*_lim_ and *J*_0_ are all in orders of Pt < MoIn_2_S_4_ < MoIn_2_S_4_@CNTs-1 < MoIn_2_S_4_@CNTs-3 < MoIn_2_S_4_@CNTs-2, suggesting that the catalytic activity of the MoIn_2_S_4_@CNTs-2 CE has enhanced greatly after doping CNTs. Among above CEs, the MoIn_2_S_4_@CNTs-2 CE gains the best catalytic activity compared to the others. The largest *J*_lim_ and *J*_0_ of the MoIn_2_S_4_@CNTs-2 CE can be ascribed to its large specific surface area made from hedgehog ball structure and enhanced conductivity by CNTs doping.

### Photovoltaic performance of the DSSCs

For contrast, the DSSCs with the MoIn_2_S_4_, MoIn_2_S_4_@CNTs, and Pt CEs are prepared with the uniform photoanodes and electrolyte. The *J*-*V* characteristic curves are measured under 1 sun (AM 1.5 G, 100 mW cm^−2^) and the corresponding photovoltaic parameters values are listed in Table 3. Four key parameters including short-circuit current density (*J*_sc_), open-circuit voltage (*V*_oc_), fill factor (*FF*), and power conversion efficiency (*η*) are usually adopted to assess the photovoltaic performance of the DSSCs. The *FF* and *η* of the DSSCs are calculated according to the Eqs. (1) and (2):

1 $$ \upeta\ \left(\%\right)=\frac{\mathrm{Vmax}\times \mathrm{Jmax}}{\mathrm{Pin}}\times 100\%=\frac{\mathrm{Voc}\times \mathrm{Jsc}\times \mathrm{FF}}{\mathrm{Pin}}\times 100\% $$

2 $$ FF=\frac{V\max \times J\max }{V\mathrm{oc}\times J\mathrm{sc}} $$

**Table 3:** Photovoltaic parameters of DSSCs

CEs	*J*_sc_ (mA cm^−2^)	*V*_oc_ (V)	*FF*	*η* (%)
CNTs	12.57	0.745	0.387	3.62
MoIn_2_S_4_	14.46	0.760	0.677	7.44
MoIn_2_S_4_@CNTs-1	16.31	0.744	0.672	8.16
MoIn_2_S_4_@CNTs-2	17.17	0.745	0.655	8.38
MoIn_2_S_4_@CNTs-3	16.67	0.773	0.644	8.31
Pt	15.48	0.754	0.686	8.01

where *P*_in_ is the incident light power and *J*_max_ (mA cm^–2^) and *V*_max_ (*V*) are the current density and voltage at the point of maximum power output in the *J–V* curves, respectively.

**Fig. 8:**
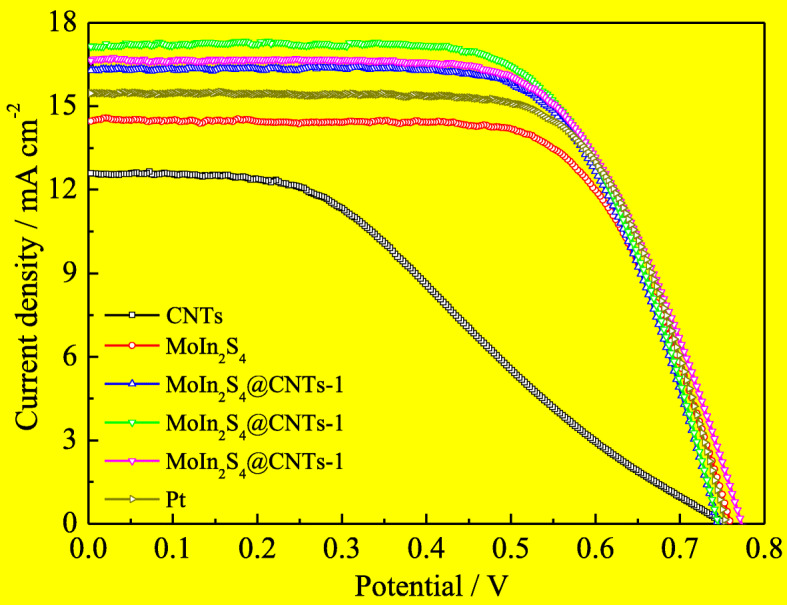
*J*-*V* characteristics of the DSSCs fabricated with different CEs

As can be seen from Fig. 8, the DSSCs with MoIn_2_S_4_ and CNTs CEs have power conversion efficiency of 7.44% and 3.62%. Compared to the DSSCs with the MoIn_2_S_4_ and CNTs CEs, the DSSCs assembled with the three MoIn_2_S_4_@CNTs CEs exhibit improvement *J*_sc_ and *η* values. Moreover, the *J*_sc_ and *η* values of the DSSCs based on the MoIn_2_S_4_@CNTs CEs increase with the CNT contents increasing from 10 to 20 mg. While further increases the CNT content to 30 mg, it results in a slight descent for the *J*_sc_ and *η*. Compared with the Pt-based DSSC, all the three MoIn_2_S_4_@CNTs-based DSSCs exhibit improved *η* of 8.16%, 8.31%, and 8.38%, which are higher than that of the Pt-based DSSC (*η* of 8.01%) under the same condition. Especially, the DSSC assembled with the MoIn_2_S_4_@CNTs-2 CE shows the best photovoltaic performance and achieves a *η* of 8.38%, and its corresponding *J*_sc_ of 17.17 mA cm^−2^, *V*_oc_ of 0.745 V, and *FF* of 0.655. The improved photoelectric property of the DSSC with the MoIn_2_S_4_@CNTs-2 CE was due to the synergetic effect of the CNTs and the MoIn_2_S_4_.

## Conclusions

Ternary MoIn_2_S_4_ and MoIn_2_S_4_@CNTs counter electrodes are fabricated on FTO substrate by using a facile one-step hydrothermal method and served in DSSCs. Under optimal conditions, the DSSCs based on the MoIn_2_S_4_@CNTs CEs all achieve good power conversion efficiency. Especially, the DSSC with the MoIn_2_S_4_@CNTs-2 composite CE exhibits a good power conversion efficiency of 8.38%, which is much higher than that of the DSSCs with the MoIn_2_S_4_ CE (7.44%) and the Pt electrode (8.01%). The enhanced photoelectric property of the DSSC with the MoIn_2_S_4_@CNTs-2 CE was due to the synergetic effect of the CNTs and the MoIn_2_S_4_. Meanwhile, the synergetic effect of the MoIn_2_S_4_@CNTs CE in electrochemical performance has confirmed from a series of electrochemical test including cyclic voltammetry, electrochemical impedance, and Tafel curves. The composite MoIn_2_S_4_@CNTs film possesses large specific surface area through N_2_ adsorption-desorption isotherms test, which is advantageous to adsorb more electrolyte and provide larger active contact area for the electrode. The facts of the MoIn_2_S_4_@CNTs CE served in DSSC broadens the potential applications of transition metal complex semiconductors in the field of optoelectronic chemistry.

## Data Availability

All data sets on which the conclusions of the manuscript rely are presented in the main paper.

## Abbreviations

CE: Counter electrode
CV: Cyclic voltammetry
DSSC: Dye-sensitized solar cell
I^−^/I_3_^−^: Iodide/triiodide
*J*_0_: Exchange current density
*J*_lim_: Limiting current density
*J*_ma*x*_: Maximum current density
*J*_sc_: Short-circuit current density
*J-V*: Photocurrent-photovoltage
*P*_in_: Incident light power
*R*_ct_: Charge transfer resistance
*R*_s_: Series resistance
SEM: Scanning electron microscopy
*V*_max_: Maximum voltage
*V*_oc_: Open-circuit voltage
